# Vitamin D Deficiency and Sarcopenia in Older Persons

**DOI:** 10.3390/nu11122861

**Published:** 2019-11-21

**Authors:** Francesca Remelli, Aurora Vitali, Amedeo Zurlo, Stefano Volpato

**Affiliations:** Department of Medical Sciences, University of Ferrara, 44121 Ferrara, Italy; rmlfnc1@unife.it (F.R.); vtlrra@unife.it (A.V.); amedeo.zurlo@unife.it (A.Z.)

**Keywords:** Vitamin D, aging, physical frailty, malnutrition, sarcopenia, nutritional intervention

## Abstract

Vitamin D deficiency is a common health problem worldwide, in particular among older people. Vitamin D regulates and modulates the physiology and function of multiple human systems, including the skeletal muscle. The effect of vitamin D on the muscle has been widely investigated, suggesting that this hormone can stimulate the proliferation and differentiation of skeletal muscle fibers, maintaining and improving muscle strength and physical performance. Older persons have a higher prevalence of low Vitamin D levels as a consequence of low dietary intake and reduced ultraviolet irradiation of the skin. Therefore, older people with vitamin D deficiency might be at risk of sarcopenia, a geriatric syndrome characterized by the progressive loss of skeletal muscle mass and strength often complicated by adverse events, such as falls, disability hospitalization and death. Several randomized clinical trials have been conducted to investigate the effect of oral vitamin D supplementation in older patients to prevent or treat sarcopenia, but results are still controversial. In this narrative review we summarize the biological, clinical and epidemiological evidence supporting the hypothesis of a causal association between Vitamin D deficiency and an increased risk of sarcopenia in older people.

## 1. Introduction

**Figure 1:**
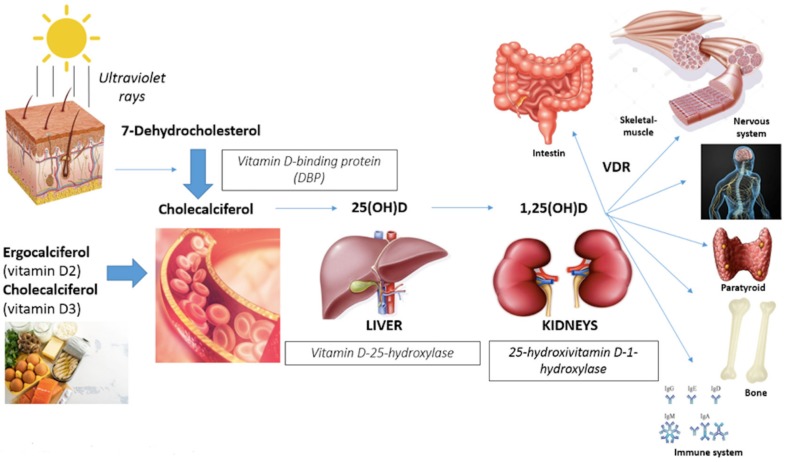
Effects of Vitamin D and target organs.

Vitamin D, a fat-soluble vitamin, is a hormone supplied by cutaneous synthesis on sunlight exposure (90%) and dietary intake (10%) [1,2]. The precursor of vitamin D is 7-dehydrocholesterol (pro-vitamin D3), which is synthesized by the liver from cholesterol, and it is converted in the skin first to pre-vitamin D3, then to cholecalciferol (vitamin D3) by solar energies under the ultraviolet ray’s action [3,4] (Figure 1). The endogenous production of vitamin D depends on age, ethnicity, availability of the precursor in the skin, skin pigmentation, seasonal variation of sun luminosity, regional latitude, daytime and duration of sun exposure, skin area exposed, use of sunscreen and clothing [5,6,7]. The dietary source is constituted by ergocalciferol (vitamin D2), present in vegetables (for example some types of mushrooms called shiitake), and cholecalciferol that is abundant in naturally rich animals foods like eggs, cod liver oil, fish fat, such as salmon, sardines, mackerel and tuna [8], and some fortified foods including milk, juices and cereals [9].

In the bloodstream, vitamin D is bound to the vitamin D-binding protein (DBP) that carries it to the liver where it is metabolized into 25-hydroxyvitamin D (25(OH)D) or calcidiol, the biologically-inactive form, by the vitamin D-25-hydroxylase enzymes. Then it is transported to the kidney where it is metabolized to the biologically-active form 1,25-dihydroxyvitamin D (1,25(OH)2D) or calcitriol by the enzyme 25-hydroxyvitamin D 1-hydroxylase or 1-alpha-hydroxylase (1-OHase) [10].

Several mechanisms regulate the Vitamin D metabolism, including a self-regulated negative feedback and others, depending on serum phosphate and calcium levels, fibroblast growth factors (FGF-23) and parathyroid hormone (PTH) [11]. Moreover, other hormones, acting on PTH, can regulate calcitriol synthesis, among these: glucocorticoids, estrogens, calcitonin and somatotropin. 

Lastly, vitamin D regulates its serum levels with autoregulation mechanisms, inhibiting the anabolic enzyme 1-OHase and enhancing the catabolic enzyme 24-OHase (direct regulation) and inhibiting PTH gene transcription (indirect regulation) [12].

1,25(OH)D, the biologically-activated form of vitamin D, binds to the nuclear vitamin D receptor (VDR) that forms heterodimers with related receptors and binds to vitamin D response elements (VDREs) to initiate intracellular signaling cascades affecting gene expression [13]. VDR expression depends on age, gender and pathology; moreover, it has been reported that there are also VDR genotypic variations, correlated to differences in muscle strength [6]. Vitamin D receptors are nuclear receptors present in many organs [5] and promoting different actions (Figure 1):

- Gastrointestinal track (calcium absorption);
- Bone (induction of bone remodeling turnover with calcium deposition in newly-formed bone);
- Immune cells system (anti-inflammatory effects with suppression of interleukine-6 and neoplastic cells proliferation);
- Myocardium, vascular smooth muscles and endothelium (remodeling cardiac muscle and improving in flow-mediated dilatation and blood pressure);
- Nervous system (affecting neuronal differentiation, maturation and growth, neuroplasticity and neurotransmission);
- Musculoskeletal system (proliferation and differentiation muscle fibers) [6,14].

To determine vitamin D status in clinical practice, the inactive form 25(OH)D is measured in serum: for adults, optimal levels are above 30 ng/mL, whereas more than 40 ng/mL are suggested for older people. Insufficiency and deficiency are currently defined as vitamin D levels between 20 to 29 ng/mL and below 20 ng/mL (or 50 nmol/L), respectively [10], with these conditions being very common in older populations.

## 2. Role of Vitamin D on the Skeletal Muscle System

A relevant number of epidemiological studies have suggested the potential role of vitamin D in order to maintain or improve muscle strength and function, physical performance and preserve independence in older people [14,15]. Although, the biological role of vitamin D on skeletal muscles function has been widely investigated [16,17], in the past years the expression of VDR on skeletal muscle cells has been questioned. 

In fact, some studies, using specific and sensitive immunohistochemical assays, showed that the vitamin D receptor was undetectable in skeletal, cardiac and smooth muscle, suggesting an indirect involvement of the hormone on the muscles themselves [18]. On the other hands, subsequent studies revealed that VDR can be localized in the nucleus of human muscle cell lines, myoblasts [19] and adult skeletal muscle [20,21].

Furthermore, other studies showed that VDR expression changes over the life span, being more expressed in satellite cells than in mature muscle fibers, and being less expressed with increasing age. This would suggest a key role of vitamin D in early-stage muscle development, but a less important role in muscle physiology in older age [7,19].

Different biological mechanisms by which vitamin D might regulate skeletal muscle function have been evaluated in cellular models. There are genomic effects through the interaction between vitamin D, VDR and specific nuclear receptors that influence gene transcription, and non-genomic effects described by the interaction between vitamin D and its non-nuclear receptors, activating intracellular signal transduction by other complex pathways [22,23,24]. Functional in vitro studies have demonstrated the direct biological role of the active vitamin D form in the regulation of genes and signaling pathways affecting calcium and phosphate homeostasis, proliferation and differentiation of muscle cells [12,16]. Vitamin D induces cell proliferation through the up-regulation of follistatin and insulin-like growth factor 2. It affects cell differentiation inducing several myogenic transcription factors including fetal myosin, the neural cell adhesion molecule, B-cell lymphoma 2, insulin-like growth factor-I, fibroblast growth factor, retinoblastoma protein and myogenic differentiation protein 1. It regulates muscle regeneration initiation, promoting an increase of the cross-sectional area of skeletal muscle fibers by cell cycle arrest, an important step for myogenic initiation [10,25,26]. Vitamin D is also involved in calcium and phosphate transport across cell membranes and phospholipid metabolism. Moreover, it suppresses myostatin expression, a negative regulator of muscle, preventing muscular degeneration and improving the contractile filaments and muscle strength [27]. In a recent study Girgis et al. have demonstrated the presence of VDR in myocites in mice, and that VDR deletion was associated with reduced lean mass, sarcopenia, reduced grip strength and exercise performance, reinforcing the hypothesis of the Vitamin D–VDR interaction as key biological factor in skeletal muscle physiology [28].

### 2.1. Effects Vitamin D on Muscle Cell Types

Muscle cells can be divided in two main types: type I and type II [29].

- Type I muscle cells are considered slow twitch, characterized by aerobic metabolism with low power production and high endurance capacity. They present a thick network of capillaries, important for carrying more oxygen, and a large quantity of myoglobin and mitochondria, for fat and carbohydrates’ oxidative phosphorylation. For these reasons, they have red color. Because of their lower strength and slow speed of contraction, they are essential for endurance exercise.
- Type II muscle cells are defined as fast twitch, characterized by anaerobic metabolism with high speed and strength contraction, important for sprinting exercises. There are two major subtypes, divided according to speed and force generated: IIA identified as “fast twitch oxidative” with intermediate characteristics between type I and II (also these, in fact, have the red phenotype) and IIB defined as “fast twitch glicolytic” characterized by high power and low endurance. Only the latter have a pale color, due to a low number of mitochondria, lower amount of myoglobin and fewer capillaries.

Vitamin D affects the diameter and the number of type II muscle cells, in particular type IIA, that induce fast muscle contraction velocity, and are crucial for anaerobic maximal intensity short-burst activities such as sprinting, acceleration and deceleration, jumping and change of direction [16]. Type II fibers are important, not only for young athletes, but also for the elderly, because of their capacity to reduce, for example, the risk of falling. 

Using muscle biopsy in patients with vitamin D deficiency, Ceglia and colleagues have shown that vitamin D deficiency is associated with muscle abnormalities characterized by type II fiber atrophy, muscle infiltration with fat cells and glycogen granules, enlargement of the inter-fibrillary gaps and increased fibrosis [13,30]. Conversely, Knutsen showed that vitamin D supplementation increases the number and the diameter of type II fibers by inducing muscle protein synthesis and myogenesis with a consequent increase in muscle strength and better neuromuscular performances [9,31].

### 2.2. Epidemiological Association between Vitamin D and Muscle Strength and Physical Performance

According to current literature, vitamin D is associated with neuromuscular performance, but results on the association of vitamin D levels with muscle strength and physical performance in the older persons are somehow discordant. For example, in the Invecchiare in Chianti study (aging in the Chianti area, InCHIANTI), a prospective study performed on 1155 people ≥ 65 years randomly selected from the Chianti geographic area (Tuscany, Italy), researchers have shown a significant association between low vitamin D levels and poor physical performance, assessed by handgrip strength and the short physical performance battery (a lower-extremity objective performance based evaluation tool, including raising five times from a chair, maintaining balance in three more challenging positions and the four-meter usual walking speed test) [32]. Older adults with serum vitamin D levels less than 10 ng/mL performed worse compared with the others. On the other side, other clinical studies suggested that those who had higher vitamin D levels exhibit better lower extremity muscle performance [33,34]. Similar results were found in the Rancho Bernardo Study cohort [35]. In the longitudinal Newcastle 85+ Study, Granic et al. evaluated the association between vitamin D concentration and muscle strength and physical performance change in very old adults over 5 years of follow-up [19]. Vitamin D was categorized into seasonal-specific quartiles (SQ1–SQ4) in order to account for seasonal variation in UVB exposure and vitamin D skin production. Muscle strength was evaluated using hand grip strength, physical performance was assessed by the Timed up-and-Go test (TUG), recording the time needed to get up from a chair, walk in straight line for 3 m to and back from a marker placed on the floor and sit back on the chair. The authors found that the lowest vitamin D season-specific quartile was associated with a higher rate of muscle strength decline in men aged > 85, whereas physical performance decline (TUG) did not differ across vitamin D quartiles. Nevertheless, as mentioned, the available literature on this issue is still controversial. For example, a cross-sectional study among a cohort of community-dwelling women ≥ 75 years, did not demonstrate any relations between low vitamin D concentration and low lower limb and handgrip muscle strength, assessed with a computerized dynamometer [36]. Also, a prospective study on persons older than 80 years, published in 2013, has confirmed these findings, showing no association between serum vitamin D level and muscle strength and physical performance, assessed by means of static balance, gait speed and grip strength [37].

Although many prospective studies have examined the vitamin D role in muscle strength and physical performance in older adults [34,35,38,39,40,41,42], there is high heterogeneity in term of participants characteristics and type of muscle strength assessment, and only a few projects included very old people (aged 85 and older), despite this subgroup being at the greatest risk of low vitamin D status, muscle mass and strength loss, and functional decline [43].

## 3. Sarcopenia and Frailty

**Figure 2:**
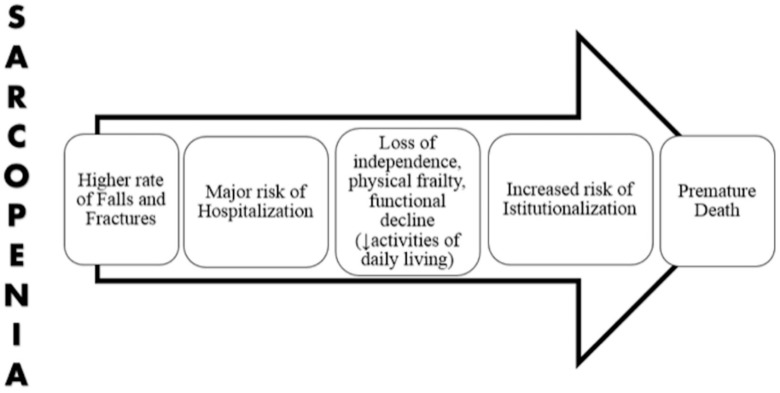
Putative pathway from sarcopenia and health outcomes.

Sarcopenia is an age-related clinical condition characterized by progressive loss of skeletal muscle mass with reduced muscle strength and physical performance [44]. It is a powerful risk factor for adverse events, including delirium [45], disability, institutionalization and death [46] (Figure 2). Nowadays, the prevalence of sarcopenia is increasing, probably related also to an increased life expectancy in developed countries. In a multicenter Italian study, published in 2017, sarcopenia that was diagnosed according to the European Working Group on Sarcopenia in Older People (EWGSOP1) criteria, has reached 36.4% in the hospitalized geriatric population, and being more common among males [47]. 

With regards to pathophysiology and changes in the architecture of skeletal muscle, sarcopenia is due to a reduction in the number and size of muscle fibers, particularly type 2, in a single motor unit with a concurrent gradual infiltration of muscle fibers by adipose and connective tissue [48].

There is not a single diagnostic definition of sarcopenia universally accepted by the scientific community. In clinical research and practice, two operational definitions are more often used; the first proposed by EWGSOP in 2010 and revised in 2018 (EWGSOP2) [49,50], and the second suggested by the FNIH Sarcopenia Project [51]. The more recent EWGSOP2 criteria distinguish three categories of sarcopenia: probable sarcopenia (when low muscle strength is detected), definite sarcopenia (with in addition low muscle quantity/quality) and severe sarcopenia (if low muscle strength and low muscle quantity/quality plus low physical performance are simultaneously present). The EWGSOP2 criteria cut-off for low muscle strength are by hand grip strength < 27 kg in men (< 16 kg in women) and chair stand > 15 s for 5 rises; the cut-off points for low muscle quantify are an appendicular skeletal muscle mass (ASM) < 20 kg in men (< 15 kg in women) and an ASM/height^2^ < 7.0 Kg/m^2^ in males (< 5.5 Kg/m^2^ in females); lastly, the EWGSOP2 cut-offs for low physical performance are a gait speed ≤ 0.8 m/s, a Short Physical Performance Battery (SPPB) ≤ 8 point score, a Timed Get Up and Go Test (TUG) ≥ 20 s and 400-m walk test not completed or ≥ 6 min [52].

The FNIH Sarcopenia Project has considered the presence of weakness and low lean mass as cornerstones of the definition. In particular, the cut-off points recommended are the following: to define weakness, it is necessary the presence of a hand grip strength < 26 kg in men and < 16 kg in women (alternatively, grip strength adjusted for Body Mass Index < 1.0 in men and < 0.56 in women); while to assess appendicular lean mass (ALM), it is recommended an ALM adjusted for BMI < 0.789 in men and < 0.512 in women (alternatively, ALM < 19.75 kg in men and < 15.02 kg in women) [53].

**Figure 3:**
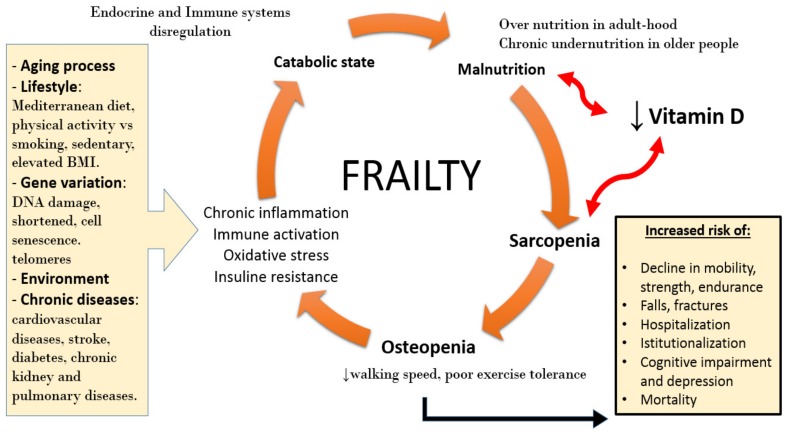
Putative role of vitamin D in sarcopenia and frailty.

Loss of mass muscle and strength are strictly connected to the concept of frailty [54], as depicted in Figure 3. In her pioneering paper, Linda Fried defined frailty as a clinical syndrome characterized by: (1) reduced muscle strength; (2) fatigue and subjective feeling of easy fatigability; (3) unintentional weight loss; (4) reduced walking speed; (5) reduced level of physical activity. With at least 3 of previous criteria, the patient was defined “frail”; while with the presence of 1 or 2 criteria, the patient was considered as “pre-frail” [55]. Whereas, according to Rockwood [56], frailty is defined as an expression of a status of vulnerability to adverse outcome (exacerbation of chronic disease, delirium, falls, disability, hospitalization, death) due to body’s damages, stored during life. 

To assess the presence and severity of frailty, Rockwood proposed a tool, called the Frailty Index (FI), defined as the ratio between the sum of all patient’s damages and the total “possible” damages (listed in a table of reference). Using the FI, the clinician can perform a multidimensional evaluation and quantify the degree of frailty: a higher score is equivalent of a major vulnerability of the patient. A third more recent definition, proposed as an alternative to identify frail patients, is the “physical frailty & sarcopenia” (PF&S) operationalized and adopted by the SPRINTT consortium [57]. PF&S identifies a new clinical condition, defined by the presence of low appendicular lean mass and functional impairment; thus, muscle atrophy becomes the biological underpinning mechanism of physical frailty. In older patients, PF&S is significantly related to a high risk of disability and other negative outcomes [58].

The biological connection between sarcopenia and frailty is hypothesized as an abnormal activation of the inflammatory system with an increase of cytokine release, a neuro-endocrinal dysregulation with high cortisol serum levels and low levels of anabolic hormones (IGF-1, GH and sexual hormones), along with a reduced level of physical activity and reduced dietary intake. All these mechanisms are implicated in sarcopenia, osteoporosis, loss of functions, and therefore, frailty. Although considered a strongly age-related condition, sarcopenia can also be due to other predisposing factors (secondary sarcopenia). Specifically, sarcopenia can be activity-related (sedentary life-style, bedridden), disease-related (organs failure, neoplasia, inflammatory chronic diseases, endocrinopathies) and nutrition-related (caloric-protein malnutrition, malabsorption). Considering this premise, it is clear that the geriatric population is extremely exposed to develop this pathological condition.

Since sarcopenia has a multifactorial etiology, clinicians can potentially act on three major aspects for prevention and treatment: physical activity, nutritional support and medication. Exercise slows and reduces the age-related loss of mass and strength of skeletal muscle [59]. Resistance (isometric) training is indicated as preventive intervention against sarcopenia; in fact, it increases the size of type 2 fibers, with a recruitment of satellite cells and reduces deposition of intramuscular adipose tissue; furthermore, isometric activity reduces insulin resistance in skeletal muscle, and systemic chronic inflammation. Physical activity has also a synergistic effect with protein and essential amino acids intake in preventing age-related loss of muscle mass and strength and preventing the onset of sarcopenia in bedridden and hospitalized patients [52,53]. 

In older patients the recommended protein intake is higher than that recommended for adults (i.e., 1.2 vs. 0.8 g per kilogram of body weight). For example, a composite nutritional intervention with oral supplements, rich of proteins, amino acids, the leucine metabolite hydroxy-beta-methylbutyrate (HMB), vitamin D, and high caloric content seems to decrease the risk of death at 90-days in hospitalized malnourished patients [60]. Finally, no drugs have shown a significant effect for the prevention and treatment of sarcopenia, and therefore no molecules have been approved by the regulatory agencies for this condition [61].

## 4. Vitamin D Deficiency and Sarcopenia

In the older population, vitamin D deficiency is common and widespread all over the world. Older patients are particularly prone to develop vitamin D insufficiency or deficiency for several reasons, including a reduced cutaneous synthesis and sun daily exposure or various diseases, as chronic renal failure or gastrointestinal malabsorption [10,58].

Vitamin D binds to the VDR receptor on muscle fibers and increases their size, improving muscle strength and physical performance [30]. In vivo experiments have highlighted the role of VDR receptors: in VDR null mice, the size of muscle fibers was significantly lower than wild type mice; after 8 weeks of age, the loss of muscle mass was faster in the first group than in the second one, suggesting the central functional and trophic role of VDR receptor on muscle fibers [62]. During the aging process, the number of VDR receptors on muscle tissue is progressively reduced, with a lower functional response to vitamin D, with a consequent loss of muscle mass and muscle strength [63].

Many prospective studies have examined the vitamin D role in muscle strength and physical performance in older adults [34,35,38,39,40,41,42], although they differ in participants characteristics and type of muscle strength assessment. Several studies have shown as in older people, that serum levels of vitamin D are independently related to the loss of muscle mass and muscle strength decline [34,64], more in men than in women [17,65], suggesting that older people with vitamin D deficiency are extremely exposed to develop sarcopenia. A prospective study published in 2017 has shown that low circulating vitamin D levels are associated to an accelerated loss of muscle strength (measured as hand grip strength) in men ≥ 85 years; conversely, no significant differences have been found in physical performance, assessed by TUG, over time [66]. Besides sarcopenia, observational studies suggest that older individuals with vitamin D deficiency have a major risk of other important geriatric outcomes, such as frailty and falls [62,67]. Overall, observational studies and mechanistic experiments support a biological link between a low vitamin D level and the age-related decline in muscle mass and muscle quality, suggesting that vitamin D supplementation might represent an effective way to prevent and treat sarcopenia, frailty and their clinical complications. Despite the fact that the “oldest-old” (aged > 85) are at the greatest risk of vitamin D deficiency, sarcopenia, and functional decline, only a few studies have specifically focused on this population [49]. Other prospective studies in this age group are needed to corroborate vitamin D for muscle-skeletal health in later life.

## 5. Nutritional Intervention

Concerning the prevention and treatment of sarcopenia, while the efficacy of caloric and protein oral supplementation is universally accepted [30,62,63], the role of vitamin D supplementation in deficiency states is still controversial. Several studies, summarized in Table 1, have investigated the effect of oral vitamin D supplementation for the prevention of sarcopenia and frailty, yielding conflicting results [66,68,69,70,71,72,73,74].

One randomized controlled trial (RCT) published in 2009 suggested the effectiveness of vitamin D supplementation on muscle strength in a sample of institutionalized older patients [74]: daily calcium plus monthly placebo was given to the control group, while daily calcium plus monthly vitamin D (first 2 months: 150.000 IU; last 4 months: 90.000 IU) was administrated to the intervention group. After 6 months of intervention, a significant difference in hip and knee extensors strength was observed between the two groups; this result suggested how the cholecalciferol supplementation may improve lower limb muscle strength in older people, despite the absence of physical activity. In another intervention study published in 2009, community-dwelling women and men ≥ 70 years old with vitamin D serum levels < 78 nmol/l were enrolled, and a daily supplementation of calcium and vitamin D (versus calcium supplementation alone) was given. After 12 months and compared to the placebo group, the oral supplementation group demonstrated an improvement in muscle strength and physical performance with a concomitant reduction in falls rates of 27% and 39% at 12 and 20 months, respectively [75]. Moreover, in 2017 an observational cohort study also reported that vitamin D supplementation (20 μg a day) for a 6-month period in post-menopausal women with a diagnosis of osteoporosis and/or vitamin D deficiency (<30 ng/mL) was significantly correlated to an increase of appendicular muscle strength (hand grip strength and knee extension strength) and physical performance (SPPB and 4-m gait speed) with a parallel reduction in the risk of falls [76]. Furthermore, a recent multi-multicenter RCT highlighted that in older sarcopenic people, oral nutritional supplementation with protein and vitamin D is effective in increasing appendicular muscle mass, measured by bioimpedentiometry (BIA) and physical performance, assessed by a chair-stand test, independent of the level of physical activity [61]. This latter study leads to a notable conclusion, inasmuch in older people with vitamin D deficiency at the baseline, it could be necessary a longer period or a higher dose of vitamin D than adults to reach the target vitamin D concentration and the beneficial effects on the muscle. Indeed, with regard to a desirable cut-off level of circulating vitamin D, the available literature is still discordant, but generally authors support at least a concentration of 50 nmol/L in the general elderly population [76], while in frail patients a minimum of 75 nmol/L, given the major risk of falls and adverse events related to a damaged osteo-muscular system [76]. Conversely, Cummings et al. [61] affirm that a very large dose of vitamin D supplementation may also increase the risk of falls, inasmuch the strategy to practice a correct integration to maintain vitamin D levels > 30 nmol/L is still undefined by the RCT. In fact, as mentioned above, the available literature about the vitamin D supplementation in sarcopenic patients is still controversial (Table 1). An intervention study published in 2003 has shown that a single dose of cholecalciferol (300.000 UI) administrated to frail older patients neither improves physical performance nor decreases the risk of falls [66]. Moreover, a randomized study recently published and performed on sedentary older men with a vitamin D < 75 nmol/L at baseline did not found any differences in SPPB, gait speed, falls and adverse events between the group that received a daily cholecalciferol supplementation (4.000 UI), as compared to the placebo group [72]. Similarly, in 2015, another intervention study, performed in Finland on women 70 to 80 years old with at least one fall in the previous year, has not shown any protective effects of exercise and vitamin D to prevent falls; moreover, while exercise has improved muscle strength and physical performance, vitamin D has not had any significant effect [74].

Finally, another recent (2019) randomized study has confirmed these results. The study was performed on healthy people ≥ 60 years old with a vitamin D levels between 8 and 20 ng/mL; oral vitamin D supplementation (858 UI daily) was administrated to the intervention group and placebo pills to the control one. After 4 months, serum vitamin D was measured in all patients and, among the participants in the intervention group, those who presented a concentration < 28 ng/mL were given an additional vitamin D supplementation (800 UI daily) and those with ≥ 28 ng/mL were administrated an additional placebo pills. After 1 year, no differences have been found between the two groups in term of leg press power, function, strength and lean mass [73].

## 6. Conclusions

Several biological, experimental and epidemiological pieces of evidence support the hypothesis that Vitamin D supplementation would be effective in preventing and treating sarcopenia in older adults. Nevertheless, because of the high heterogeneity of the observational study and the conflicting results of RCTs, the exact role of vitamin D supplementation to prevent and treat sarcopenia is still uncertain and under investigation. Additional intervention studies are needed to clarify the effect of vitamin D supplementation on skeletal muscle and its optimal serum levels to maintain a good physical function in advanced age.

In the meantime, since Vitamin D deficiency is very common in older people, and since Vitamin D has many other fundamental biological effects beside skeletal muscle trophism, clinicians should screen Vitamin D levels in sarcopenic patients, and should advocate oral supplementation to any older person with Vitamin D deficiency of insufficiency. Furthermore, patients at risk of or with established sarcopenia should be encouraged to be involved in regular physical activity and to increase their intake of proteins and/or essential amino acids.

## Figures and Tables

**Table 1:** Studies about Sarcopenia and oral vitamin D supplementation in older people.

Author/Year	Study Design	Patient Characteristics	N Included in Analyses	Intervention	Control Group	Duration	Conclusions
Moreira-Primer et al. 2009 [74]	RCT *	Institutionalized people ≥ 60 y	56	1000 mg calcium/day + 150.000 IU vitamin D/month (after 2 months: 90.000 IU/month)	1000 mg calcium/day + placebo/month	6 months	Strength muscle: improvement of hip flexors and knee extensors strength
Pfeifer et al. 2009 [75]	RCT	Community-dwelling people ≥ 70 y with 25(OH)D ≤ 78 nmol/L	242	1000 mg calcium/day + 800 IU vitamin D/day	1000 mg calcium/day	12 months	Muscle strength and physical performance: improvement of hand grip strength and knee isometric extension strength, SPPB, TUG and 4-m walking speed
Iolascon et al. 2017 [76]	PCS	Post-menopausal women ≥ 50 y with osteoporosis and/or vitamin D deficiency	113	20 μg vitamin D/day	-	6 months	Muscle strength and physical performance: improvement of isometric leg extension strength and TUG
Verlaan et al. 2018 [61]	RCT	Sarcopenic older adults	380	20 g protein (3 g leucine) + 3 g fat + 9 g carbohydrates + 800 IU vitamin D twice daily	Iso-caloric control product twice daily	13 weeks	Muscle mass and physical performance: improvement of BIA and chair-stand test
Latham et al. 2003 [66]	RCT	Frail older people, after hospital discharge	243	Single dose of 300.000 IU	Placebo (single dose)	10 weeks	Physical performance: no improvement of quadriceps resistance exercise
Levis et al. 2017 [72]	RCT	Sedentary men 65–90 y with 25(OH)D < 30 ng/mL and SPPB ≤ 9	130	4.000 IU vitamin D/day	Placebo/day	9 months	Physical performance: no improvement of SPPB or gait speed
Shea et al. 2019 [73]	RCT	Community-dwelling people ≥ 60 y with 25(OH)D ≤ 20 ng/mL	100	858 (+800) IU vitamin D/day	Placebo/day	1 year	Lower-extremity power, strength and lean mass: no improvement of Keiser pneumatic leg press, backward tandem walk test, SPPB, dual X-ray
Uusi-Rasi et al. 2015 [74]	RCT	Home-dwelling women 70–80 y with at least 1 fall in the previous year and no use of vitamin D supplements	409	800 IU vitamin D/day ± exercise	Placebo/day ± exercise	2 years	Mass muscle, muscle strength and physical performance: no improvement of BIA and SPPB, TUG, 4-m walking speed and 5 times chair stand

* RCT: randomized controlled trial; PCS: prospective cohort study.

## Abbreviations

The following abbreviations are used in this manuscript:

1,25(OH)D	1,25-dihydroxyvitamin D
25(OH)D	25-dihydroxyvitamin D
1-OHase	25-hydroxyvitamin D-1-hydroxylase
VDR	Vitamin D receptor
VDREs	Vitamin D response elements
ASM	Appendicular skeletal muscle mass
BMI	Body mass index
BIA	Bioimpedentiometry
HMB	Hydroxymethylbutyrate
FI	Frailty Index
SPPB	Short Physical Performance Battery
TUG	Get Up and Go Test
PF&S	Physical frailty and sarcopenia
